# Modeling Skin Injury from Hot Rice Porridge Spills

**DOI:** 10.3390/ijerph15040808

**Published:** 2018-04-20

**Authors:** Torgrim Log

**Affiliations:** Department of Engineering, Western Norway University of Applied Sciences, 5528 Haugesund, Norway; torgrim.log@hvl.no; Tel.: +47-900-500-01

**Keywords:** hot porridge scalding, damage integral, thermal injury, numerical modeling

## Abstract

The present work analyzes skin burns from spills of hot rice and milk products. The traditional Norwegian rice porridge serves as an example. By testing spills on objects emulating an arm, it was concluded that spills were seldom thinner than 3 mm, and stayed in place due to the viscosity of the porridge for more than one minute. The Pennes bioheat equation was solved numerically for such spills, including heat conduction to the skin and convective heat losses from the porridge surface. Temperatures were analyzed in the porridge and skin layers, and the resulting skin injury was calculated based on the basal layer temperature. Parameters influencing burn severity, such as porridge layer thickness, porridge temperature, removal of the porridge and thermal effects of post scald tempered (15 °C) water cooling were analyzed. The spilled porridge resulted in a prolonged heat supply to the skin, and the skin injury developed significantly with time. The porridge temperature turned out to be the most important injury parameter. A 70 °C porridge temperature could develop superficial partial-thickness burns. Porridge temperatures at processing temperatures nearly instantly developed severe burns. It was demonstrated that prompt removal of the hot porridge significantly reduced the injury development. The general advice is to avoid serving porridge and similar products at temperatures above 65 °C and, if spilled on the skin, to remove it quickly. After such scald incidents, it is advised to cool the injured area by tempered water for a prolonged period to stimulate healing.

## 1. Introduction

Burn injuries are of worldwide concern. Globally, approximately 265,000 burn-related deaths occur every year. It is also experienced that burns are a common injury in all societies. These burns may be a result of hot liquid and hot food spills, contact with hot surfaces, exposure to hot gases, and thermal radiation. In a single country like Bangladesh, about 173,000 children under 18 suffer from burn injuries every year [1]. In the USA, burns result in about half a million patients seeking medical treatment at hospital emergency departments. Additionally, burns are also treated at clinics, local health centers, and by private medical offices [2]. To stimulate the healing of severe burns is difficult. Burn injury treatment therefore receives much research interest [3,4,5]. Improved knowledge about thermal skin injury development, the mechanisms involved, and possible injury limiting measures are therefore much appreciated.

A systematic approach to studying thermal skin burns was initiated during the years following the World War II. A series of seminal burn studies was then published in The American Journal of Pathology. These studies included heat transport to and through porcine skin [6], the importance of time and surface temperature in developing cutaneous burns [7], and the pathology and pathogenesis of cutaneous burns on pigs [8]. It was revealed that the degree of burns was dependent on both the temperature and exposure time. A threshold temperature for skin injury was suggested. Several researchers refer to temperature above 44 °C for causing burns [9,10]. For hot liquids, others refer to 43 °C for the onset of skin injury [11]. Skin models have also been built for simulating “skin” temperatures during controlled heat flux exposure [12]. The skin injury modeling assumes that the injury develops linearly with time and exponentially with absolute temperature, i.e., an Arrhenius type of damage development.

Human skin pain receptors are located at approximately 0.1 mm depth and the pain temperature threshold is 44.8 °C [13,14]. This is above the assumed threshold temperatures for slow injury development. However, as burns usually involve much higher basal layer temperatures, the pain signal gives a suitable warning about excessive skin surface heating. In burn scenarios, the skin heating may often be quite instantaneous, i.e., the damage develops even though the victim is warned by the ongoing process. Scalding by hot porridge and similar hot food products may also be an example of nearly instant skin heating, often well above the pain threshold temperature. The best way to reduce scald risk is to prevent hot spills. However, as hot food is an important part of most, if not all, cultures, scald burns by hot food do happen.

It is known to the research and medical communities that removing the heat source and cooling the affected area is very beneficial regarding limiting scald injury. Through statistical analysis of scald patient records involving Chinese congee (a high viscosity rice soup thermally quite similar to the Norwegian rice porridge), Lau et al. [15] revealed that prompt removal of clothing after congee scalding reduced post-burn morbidity. This straightforward act may sometimes not be made in a timely manner, due to embarrassment in e.g., restaurants, etc., as well as wrong information about the importance of heat source removal.

For some special cases, skin heat exposure may be studied using analytical mathematics [16]. In most scalding scenarios, analytic solutions are usually too constrained for covering any complicated mathematical boundary conditions. Numerical modeling, on the other hand, is flexible, allowing for analyzing the situation with realistic thermal properties of the heat source, as well as the skin layers. Blood perfusion and metabolic heat production, as well changing boundary conditions during a modeling case, may also be modeled. The analysis presented in the present work is therefore based on solving the Fourier-type heat transfer equation numerically. For the scald scenarios, this has recently been done successfully, also by others [17,18,19]. Research studies on food processing provides thermal property data necessary for modeling heat transport within hot rice and milk porridge [20].

The purpose of the present work is to analyze temperature development and skin injury for spills of hot rice and milk porridge to the skin by numerical modeling. The influence of porridge temperature, possible removal of the hot porridge, water cooling, etc., is studied, and the injury for these cases is calculated based on the basal layer temperature. Spills on the arms are used as an example. Pennes bioheat equation was used for calculating the corresponding skin injury (damage integral). The paper is unique in analyzing the whole process including heat transport in the hot porridge and in the skin layers. Skin temperatures are presented for selected cases, as well as the injury development as a function of time. Some final conclusions are drawn regarding the most influential parameters identified and possible actions that may limit the associated skin injury of such spills.

The paper starts with explaining research on burns and burn modeling (Section 1). Then, the theory of heat transfer, blood perfusion, and metabolic heat production is presented, as well as the temperature and damage integral modeling (Section 2). Then, the findings are presented (Section 3) before the modeling, and the results are discussed (Section 4) followed by the conclusions (Section 5). A strong motivation for publishing this work is to provide information about possible mitigation measures, should hot food scalding happen to any of the readers or to their next of kin.

## 2. Heat Transport and Damage Integral 

### 2.1. Heat Transport Modeling

The heat conducted in a solid may be described by a linear relationship between the temperature gradient and the heat flux, i.e., Fourier’s law:

(1) $${\dot{q}}_{k}^{\prime \prime }=-k\cdot \nabla T\;\left(W/m^{2}\right)$$

where k (W/m·K) is the thermal conductivity of the solid, i.e., in the present study the porridge and the skin. The general heat balance for bioheat transfer in the skin may be expressed by Pennes bioheat equation:

(2) $$\rho C\frac{\partial T}{\partial t}=-\nabla \cdot {\dot{q}}_{k}^{\prime \prime }+W_{b}\rho_{b}C_{b}\left(T_{b}-T\right)+Q_{met}+Q_{ext}\;\left(W/m^{3}\right)$$

where ρ (kg/m^3^) is the skin density, C (J/kg·K) is the skin specific heat, t (s) is the time, $W_{b}$ (m^3^/m^3^·s) is the blood perfusion rate, $\rho_{b}$ (kg/m^3^) is the blood density, $C_{b}$ (J/kg·K) is the blood specific heat, $T_{b}$ (K) is the supplied blood temperature, $Q_{met}$ (W/m^3^) is the metabolic heat production, and $Q_{ext}$ (W/m^3^) is heat supplied from an external heat source. In the present work, $Q_{ext}$ represents the heat supplied to the skin from the hot porridge or after porridge removal, the heat loss from the skin surface to the ambient air and to tempered water.

For short duration heat exposures, Ng and Chua [21] concluded that blood perfusion does not influence the extent of burns significantly. Lipkin et al. [22] found that about 20 s is needed for the skin to increase the blood flow. This was also recently concluded by Fu et al. [9]. In the present work, up to 60 s heat exposure is analyzed. Blood perfusion and metabolic heat production therefore needs to be included in the modeling. The blood perfusion and metabolic heat production models presented by Rai and Rai [23] were therefore included in the bioheat equation, i.e., Equation (2).

Porridge is experienced to wet human skin completely. The external heat source, i.e., the hot porridge, was therefore assumed to be in perfect contact with the skin surface. Since the diameter of the arm/thigh is much larger than the heat penetration depth, the system may be considered as a one-dimensional flat surface. This allows for studying heat flow in one dimension, i.e., only dependent on the depth (*x*-dimension). Since a moderate temperature increase is studied in the present work, it is assumed that the thermal skin properties are temperature independent.

**Figure 1:**
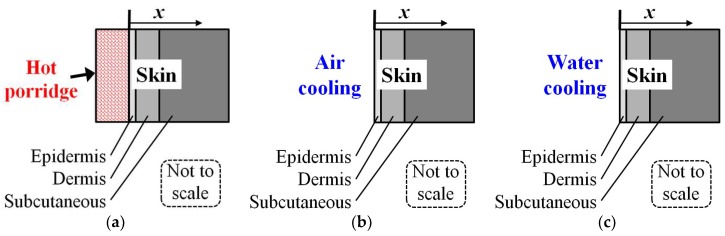
Principle sketch of the heat transfer system (**a**) during hot porridge skin contact; (**b**) after removing the porridge; and (**c**) during tempered water cooling. (*x* indicates the skin depths, i.e., for simplicity, the skin porridge interface is located at *x* = 0.)

The heat transfer model is shown in Figure 1a for the scald heating, in Figure 1b for removed porridge, and in Figure 1c for the possible final skin surface cooling by tempered water.

In the present work, it is assumed that the application of porridge to the skin surface happens instantaneously at t = 0, and that the porridge temperature at that moment is uniform. At t > 0, heat is conducted from the hot porridge into the skin, while some heat is also lost convectively from the hot porridge to the ambient air. This case is then compared to cases where the hot porridge after some time is suddenly removed, exposing the skin surface to ambient air. For some cases, the skin is also finally exposed to tempered water for post scald cooling.

To correctly determine the external heat flux to the skin surface, the heat equation must also be solved for the hot porridge layer in contact with the skin surface. For simplicity, it is conservatively assumed that the porridge wets the skin completely, i.e., there is no heat transfer barrier like air bubbles between the porridge and the skin. The representative values for thermal conductivity, density, and specific heat of rice and milk products given by [20] was used for modeling porridge heat transport in the present work, i.e.:

(3) $$k_{p}=-6.0\times 10^{-6}T^{2}+0.0015\;T+0.5061\;(W/m\cdot K)$$

(4) $$\rho_{p}=-3.4\times 10^{-3}T^{2}+0.0377\;T+1046.6\;(kg/m^{3})$$

(5) $$C_{p}=-4.0\times 10^{-3}T^{2}+0.2000\;T+3743.5\;(J/kg\cdot K)$$

The outer porridge surface at temperature $T_{p,s}$ (°C) will be convectively cooled by the ambient air at temperature $T_{\mathrm{air}}$ (°C). This heat loss may be expressed by

(6) $${\dot{q}}_{\mathrm{air}}^{\prime \prime }=h_{\mathrm{air}}\cdot \left(T_{p,s}-T_{\mathrm{air}}\right)\left(W/m^{2}\right),$$

where $h_{\mathrm{air}}$ (W/m^2^·K) is the porridge surface to air convective heat transfer coefficient. The cooling of the skin when exposed to ambient air after porridge removal was calculated by Equation (3), where the porridge surface temperature ($T_{p,s}$) was substituted with the skin surface temperature, $T_{s,s}$ (°C). For the cases where the skin was cooled by water at temperature $T_{w}$ (°C), and convective heat transfer coefficient $h_{w}$ (W/m^2^·K), the heat loss was similarly given by

(7) $${\dot{q}}_{w}^{\prime \prime }=h_{w}\left(T_{s,s}-T_{w}\right)\left(W/m^{2}\right)$$

In the present work, $h_{\mathrm{air}}$ and $h_{w}$ were estimated to be 10 W/m^2^·K and 600 W/m^2^·K, respectively. The cooling water temperature was set to 15 °C.

Solving Equation (2) numerically makes it possible to successively alter the boundary conditions for heat transport to the skin, i.e. by (i) conduction from the hot porridge in the first period, $t_{p}$ (s), (ii) air cooling for a period $t_{a}$ (s) after removing the porridge, and finally (iii) cooling the skin by tempered water at $t_{w}$ (s). The skin temperature was initially, i.e., at t=0, set to 37 °C [11] for all depths 0 ≤ x ≤ Δ, where Δ (m) represents the modeling domain size. The domain included the skin layers shown in Table 1. For the period of contact with a hot porridge layer of thickness L (m), the boundary condition to the air surface at x=−L is given by:

$$k\frac{\partial T}{\partial x}=h_{\mathrm{air}}\left(T_{\mathrm{air}}-T\left(-L,t\right)\right)\;\mathrm{for}\;0\;<\;t\;\leq \;t_{p}$$

At time $t_{p}$, the hot porridge is instantaneously removed, and the skin surface at x=0 is then exposed to ambient air, with the following boundary condition:

$$k\frac{\partial T}{\partial x}=h_{\mathrm{air}}\left(T_{\mathrm{air}}-T\left(0,t\right)\right)\;\mathrm{for}\;t_{p}\;<\;t\;\leq \;t_{w}$$

For selected cases, at time $t_{w}$, the final cooling of the skin surface by tempered water is started, with the following boundary condition:

$$k\frac{\partial T}{\partial x}=h_{w}\left(T_{w}-T\left(0,t\right)\right)\;\mathrm{for}\;t\;>\;t_{w}$$

The inner skin surface boundary condition, i.e., at x=Δ, is for simplicity given as the contact with an adiabatic surface, i.e.,

$$k\frac{\partial T}{\partial x}=0\;\mathrm{for}\;\mathrm{all}\;t\;\mathrm{at}\;x=\Delta .$$

The domain size (depth of the skin) must be sufficiently large to limit any influence of the finite dimensions. A depth ∆ > $2\sqrt{at}$ [24] would normally be sufficient to ignore the influence of the finite domain size, where t (s) is the time, and a (m^2^/s) is the thermal diffusivity given by

(8) $$a=\frac{k}{\rho C}\;(m^{2}/s)$$

In the present work, the muscle layer was included in the calculation domain, even though this was not needed to comply to the ∆ > $2\sqrt{at}$ criterion.

The literature values for skin layer thicknesses and thermal properties vary. A comprehensive summary of skin properties relating to scalds was presented by Johnson et al. [11]. Their reported values vary somewhat from those reported by Millington and Wilkinson [25]. A typical value for the skin epidermis layer in these references is 60 μm. Since the epidermis thickness varies with the location even on one extremity, and children are known to have thinner skin, 40 μm and 50 μm was also studied in the present work.

For numerical stability during the modeling, the Fourier number must satisfy

(9) $$Fo=a\times \frac{\Delta t}{\Delta x^{2}}<0.5,$$

where Δt (s) is the numerical integration step length and Δx (m) is the numerical layer thickness. A C++ computer program was used to solve Equation (2) for the boundary conditions involved in a series of cases explained in detail in the results section. A layer thickness Δx = 10 μm and a numerical step length Δt = 2 × 10^−4^ s complied with Equation (9), also for the high thermal diffusivity muscle layer. 

### 2.2. Skin Damage Modeling

Burns and scalds were originally classified as first-degree, second-degree, and third-degree burns, according to burn severity. This classification system is still popular among laypeople. Medical personnel do, however, classify burns based on the injury depth. This includes four categories [19]: (1) Superficial (S) burns confined to the epidermal layer and characterized by slight edema and fast healing; (2) Superficial partial-thickness (SP) burns that extend into the outer part of the dermal layer and result in moderate edema but little or no scarring (typically less than 1 mm burn depth); (3) Deep partial-thickness (DP) burns that extend well into the dermal layer and are slow to heal (typically 1 mm or greater burn depth) and result in hypertrophic scarring; and (4) Full-thickness (FT) burns that extend through the entire dermis requiring skin grafting (burn depths typically greater than 2 mm). Category S burns correspond to the commonly used term first-degree burns. Category SP and DP burns correspond to second-degree burns, and category FT burns correspond to third-degree burns.

Cell injury develops due to excessive skin temperatures and resulting protein breakdown. Collagen is one of the main proteins involved. The cell injury can be calculated to evaluate the burn severity at certain depths, and is expressed by the damage index Ω [26]:

(10) $$\Omega \left(\tau \right)=-\ln\left(\frac{C_{\tau }}{C_{0}}\right)$$

where $C_{0}$ and $C_{\tau }$ represent the number of undamaged cells prior to and after the heat exposure, respectively. A damage index of 0.1 corresponds to 90% of the cells still being undamaged. A damage index of 1.0 indicates that only 36% of the cells are still undamaged. The rate of the developing skin injury can be calculated by an activation energy-based model, originally developed by Henriques [27]:

(11) $$\frac{\partial \Omega }{\partial t}=P\;\exp\left(-\frac{\Delta E}{RT}\right),$$

where P (1/s) is the pre-exponential frequency factor, ΔE (J/mol) is the activation energy, R (8.314 J/mol·K) is the molar gas constant, and T (K) is the absolute temperature. Literature data for these damage parameters vary considerably [11]. In the present work, the original data presented by Henriques and Moritz [6,27], i.e., P = 3.1 × 10^98^ 1/s and ΔE = 6.28 × 10^8^ J/mol, was used for modeling the skin injury. 

The total cell damage is obtained by integrating Equation (11) over the time interval for which the basal layer temperature is above the injury threshold temperature, i.e., 43.0 °C:

(12) $$\Omega =\int_{0}^{t}P\;\exp\left(-\frac{\Delta E}{RT}\right)\mathrm{dt}$$

Since the damage index is calculated as an integral, it is often referred to as the damage integral. A damage integral Ω = 0.53 at the basal layer was reported by Ye and He [28] as the limit for *superficial* burns and Ω = 1.0 as the limit for *superficial partial-thickness* burns. The numerical integration of Equation (12) was in the present work done in parallel to the temperature modeling, i.e., in the previously mentioned C++ program.

### 2.3. The Hot Porridge Spill Situations to be Analyzed

Practical tests with traditional Norwegian rice and milk porridge (No: risengrynsgrøt) revealed that spills could be of 2 mm thickness and upwards. A 3 mm thick and 70 °C hot porridge layer, instantaneously spilled on the forearm and not removed, represented the base case for burn injury calculations. The forearm base case epidermis thickness was set to 60 μm [11]. For comparison, the injury development was calculated for cases where the porridge was removed and water at 15 °C was applied to the burn for wound cooling. Injury development was also calculated for selected cases where epidermal thickness, porridge thickness, and initial porridge temperature were varied. The various cases are presented in the Results section.

## 3. Results

**Figure 2:**
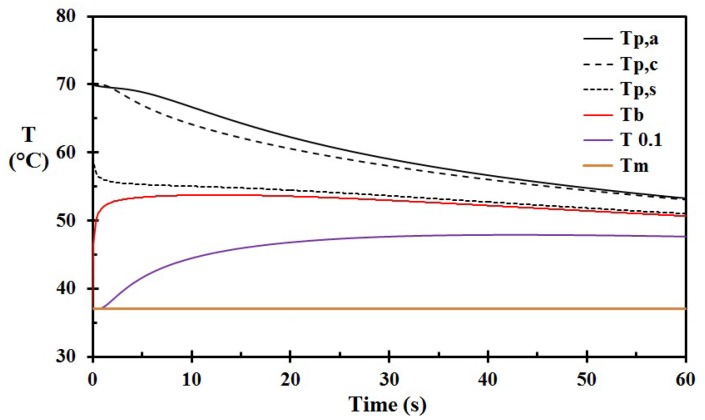
Temperatures for Case A versus time: T_p,a_: outer porridge layer in contact with ambient air; T_p,c_: center porridge layer; T_p,s_: porridge layer in contact with the skin; T_b_: basal layer temperature; T_0.1_: center of the dermis; and T_m_: center of the muscle layer. (The porridge was not removed.)

**Figure 3:**
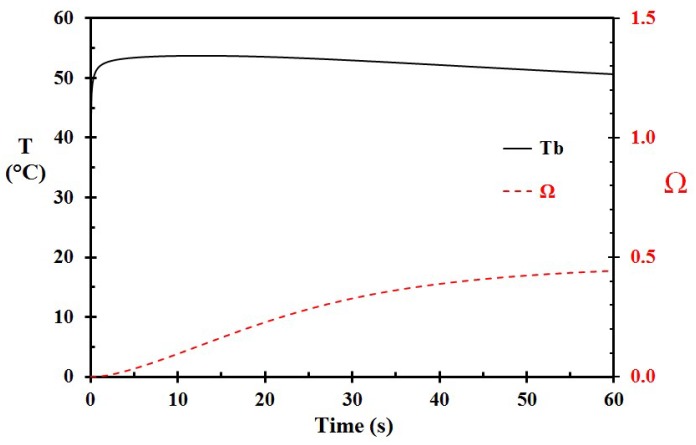
A close-up of the basal layer temperature (T_b_) and the corresponding damage integral (Ω) as a function of time for Case A, i.e., 70 °C hot porridge. (The porridge was not removed.)

The base case (Case A) was modeled with an epidermis thickness of 60 μm and a 3 mm thick porridge layer at 70 °C spilled to the skin and left on the skin for 60 s. The temperature versus time for selected depths, including the porridge, for this case (Case A), is shown in Figure 2. A close-up of the basal layer temperature and the corresponding injury development is shown in Figure 3. At 60 s, the damage integral was 0.444. It did, however, also develop until 240 s (not shown in Figure 2), reaching a maximum of 0.476. According to Figure 3, the damage integral develops nearly linearly for the first 20 s, and then gradually levels off towards a constant value. This is, to some extent, in contrast to the results obtained from scalding with hot water, where the first 5 s period is most critical for injury development [17,29]. Another major difference is that the rather low temperature of 70 °C results in damage close to the limit associated with superficial partial thickness burns, i.e., close to Ω = 0.53. This temperature is in agreement with the results reported for typical beverage spills [17,20]. This is due to the 3 mm thick porridge layer keeping the skin temperatures well above the threshold of heat damage for a prolonged period.

**Figure 4:**
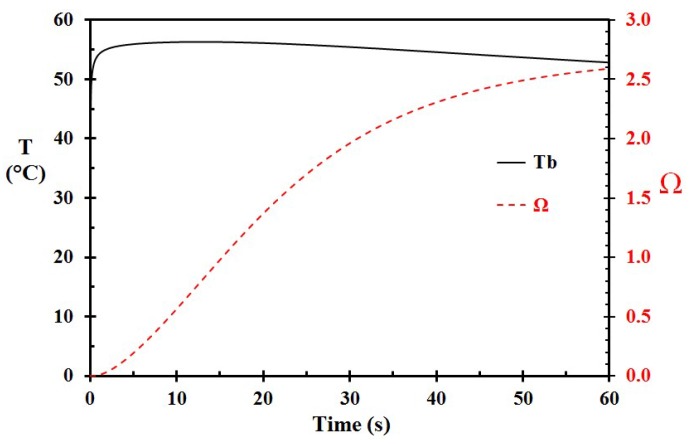
A close-up of the basal layer temperature (T_b_) and the corresponding damage integral (Ω) as a function of time for Case B, i.e., 75 °C hot porridge. (The porridge was not removed.)

For Case B, the parameters were similar to the base case (A), except that the initial porridge temperature was set to 75 °C. This resulted in a damage integral of 2.59, as seen in Figure 4. This represents a significantly more severe burn. Case C was similar to Case A (and Case B), but with an initial porridge temperature of 65 °C. This reduced the damage integral to 0.075, indicating that 65 °C is a much safer temperature for rice porridge than e.g., 70 °C and 75 °C.

Reducing the epidermis thickness from 60 μm to 50 μm (Case D) and 40 μm (Case E) increased the damage integral at 60 s from 0.444 to 0.491 and 0.549, respectively. This indicates that persons with thinner epidermis, e.g., children, are more at risk of developing burns than adults.

**Figure 5:**
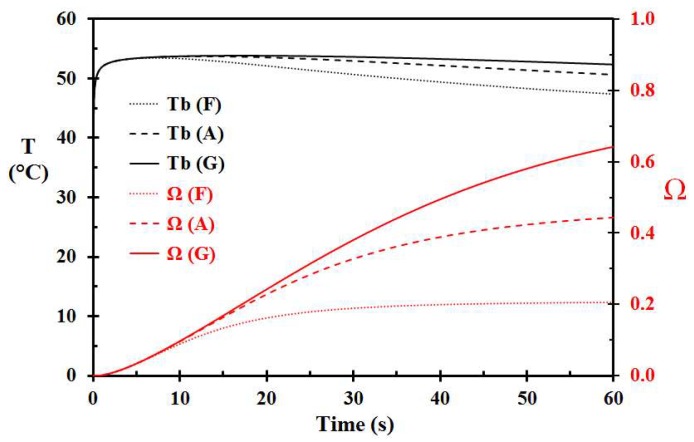
Basal layer temperature and damage integral as a function time for 2 mm (Case F), 3 mm (Case A), and 4 mm (Case G) thick layer of porridge at 70 °C spilled on the skin.

Still using the same parameters as the base Case A, reducing the porridge layer thickness to 2.0 mm (Case F), or increasing the layer thickness to 4.0 mm (Case G), respectively, changed the damage integral at 60 s from 0.444 to 0.206 and 0.642, as seen in Figure 5. This shows that the porridge layer thickness is of major importance regarding skin injury development over time. It is, however, seen in Figure 5 that these porridge layer thicknesses result in about similar skin injury for the first 10 s exposure time.

Results from different cases, e.g., removal of the hot porridge, application of cooling water, different epidermal thickness, etc., are presented in Table 2 to study the influence of these parameters on the calculated skin injury.

**Figure 6:**
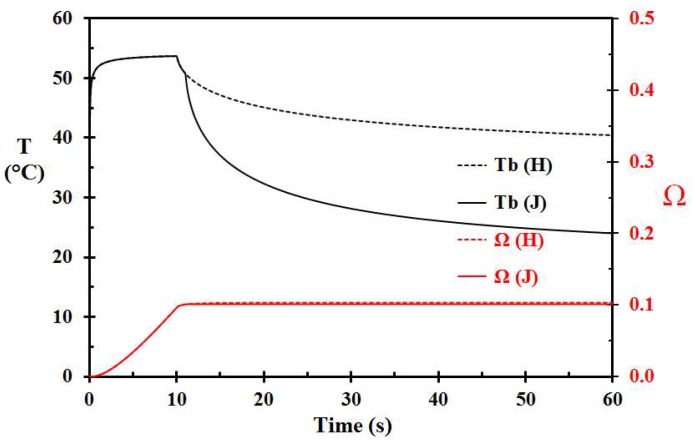
Basal layer temperature and damage integral development for Case H (porridge removal after 10 s and no water cooling) and J (porridge removal after 10 s and water cooling 1 s later).

Removal of the hot porridge at 10 s (Case H) reduced the damage integral to 0.103, as seen in Figure 6. Applying tempered (15 °C) water 10 s later (Case I) for skin surface cooling did not reduce the damage integral. Applying water 1 s after porridge removal (Case J) reduced the damage integral only marginally, i.e., from 0.103 to 0.101, as seen in Figure 6. It is clearly seen that cooling by tempered water has a limited effect regarding the modeled skin damage development.

Compared to Case H, removal of the 70 °C hot porridge after 20 s (Case K) or 30 s (Case L) increased the damage integral to 0.239 and 0.338, respectively. It may therefore be concluded that after a spill to the skin, removal of the heat source, i.e., the hot porridge, is vital regarding injury control.

**Figure 7:**
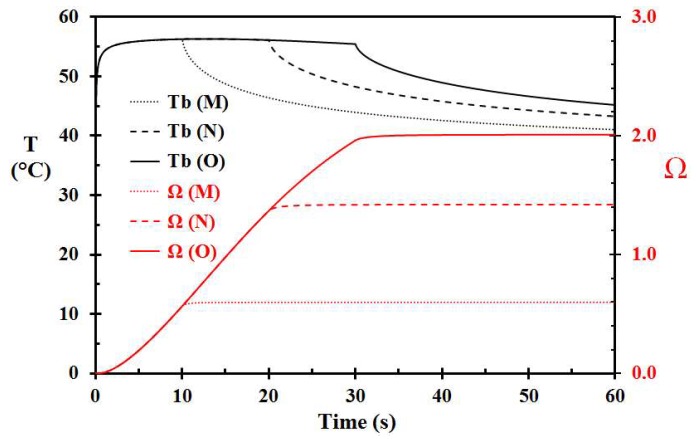
Basal layer temperature and damage integral development for 75 °C hot porridge. Case M, N, and O refer to porridge removal at 10, 20, and 30 s, respectively.

Given a 5 °C higher porridge temperature, i.e., 75 °C, removal of the porridge at 10 s (Case M), 20 s (Case N), and 30 s (Case O), respectively, gives damage integrals 0.599, 1.421, and 2.009, as seen in Figure 7. This may be directly compared with the corresponding values of 0.101, 0.239, and 0.338 for 70 °C hot porridge. This clearly shows that 5 °C warmer porridge results in significantly increased skin injury. Cooling the porridge 1 s after removal of the porridge here also gave a minor reduction in skin damage, i.e., decreasing from 2.009 (Case O) to 1.985 (Case P). Again, the water cooling reduced the injury much less than heat source removal.

**Figure 8:**
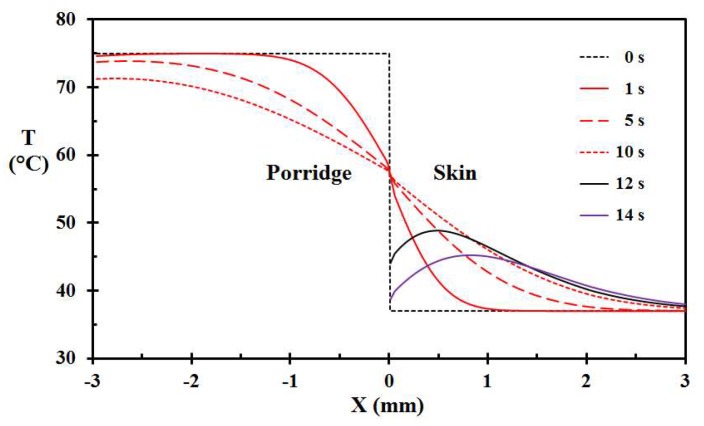
Temperature as a function of location for selected times of Case M, i.e., 75 °C hot porridge, porridge removal at 10 s, and applying water at 15 °C at 11 s.

The temperature development for 75 °C hot porridge removed after 10 s and cooled by water 1 s later (Case Q), as a function of depth is shown in Figure 8 for selected times. It is interesting to notice that the surface temperature of the skin is kept nearly constant during the first 10 s period. It only drops significantly after application of cooling water. Based on Figure 8, it looks like the porridge layer acts as a semi-infinite layer up to about 5 s. Then, the whole porridge layer temperature starts to decrease, mainly due to inward heat losses to the skin. 

**Figure 9:**
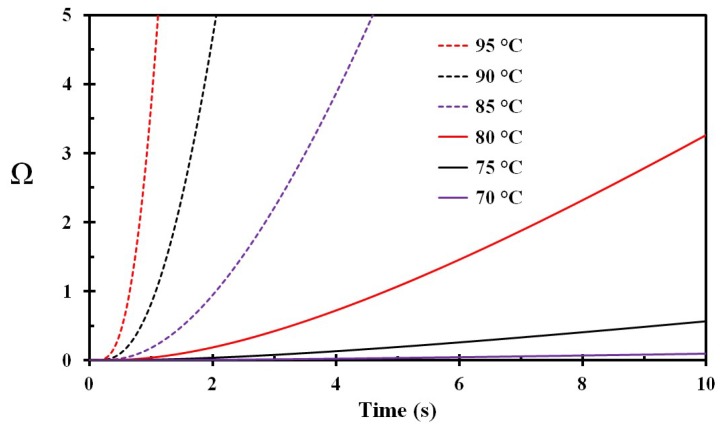
Damage integral as a function of time for spilled porridge at different temperatures (marked on the figure).

During preparation of milk and rice products, the temperatures are close to 100 °C. During such food processing, children are especially at risk of catching a porridge pot handle and pouring the hot food product over themselves. This involves spill of temperatures well above 70 °C and 75 °C, as presented in Case A and Case B. The calculated skin injury for such hot porridge are shown in Figure 9. It is clearly seen that such spills may develop very severe burns nearly instantaneously, and usually far earlier than a grown up can help in removing the hot porridge. Such spills therefore represent a major threat, especially to small children.

## 4. Discussion

A numerical modeling technique used by other researchers, e.g., [11,17,18,19], was further refined to include hot rice and milk porridge instantaneously exposing skin, i.e., imitating spilled porridge. The temperature was modeled in both the hot porridge and in the skin layers. The model allowed for changing boundary conditions throughout the modeling period, such as removing the porridge and exposing the skin to ambient air, as well as introducing tempered water cooling post scalding. The influence of other relevant parameters, such as varying epidermal thickness and spilled porridge temperature and porridge layer thickness, was also modeled. The flexibility of the numerical model provided valuable information about the skin temperatures and the corresponding skin injury development. The modeling provided knowledge regarding the potential factors influencing the hot porridge skald injury, such as removal of the hot porridge and subsequent water application to the affected skin.

For simplicity, the initial skin temperature was set to 37 °C. This conservative skin temperature has also been used by others [11]. The benefit of this simplification is that one does not need to introduce an initial skin temperature gradient.

When spilling products like rice and milk porridge, the spill represents a significant heat source, due to the low viscosity which results in comparably thick spill layers that prevent spill run off. Tests with traditional Norwegian rice and milk porridge revealed that spills could be from 2 mm thickness and thicker. The base case thickness selected for modeling in the present work was 3 mm. Spill thicknesses of 2 mm and 4 mm were also modeled. It was revealed that the skin temperature and damage development for 2, 3, and 4 mm thickness was quite similar during the first 10 s. From there on, the larger thicknesses resulted in significantly more severe injury development. 

Porridge spilled to the skin may stick to the skin for a prolonged period. Due to the thermal capacity of the porridge layer, it was demonstrated that temperatures as low as 70 °C may give skin injury associated with superficial partial-thickness burns, even for skin with normal epidermal thickness, i.e., 60 μm. Persons with thin epidermal thickness, and persons with low mobility, may be severely at risk when food products like rice and milk porridge are spilled onto the skin. It is also found that for thinner epidermis layers, the scald injury is more severe. This result is in agreement with the findings by e.g., Ng and Chua [21]. Children, who have about 30% thinner skin than adults, are therefore even more at risk [15,30]. 

Increasing the porridge temperature to 90 °C and 95 °C, the spill nearly instantaneously resulted in severe scald injury. This situation may be representative of a child grabbing a pot handle and pouring hot porridge over himself/herself. For the highest temperatures, severe injury develops long before adults may come to assistance and remove the spill. The larger the area involved, the longer time it will take to remove the spill completely. One may conclude that children are especially at risk when it comes to severe hot porridge scald injuries.

It was clearly demonstrated that the porridge temperature is of great importance regarding development of hot porridge scald injury. It was also demonstrated that fast removal of the hot porridge was the most important injury control factor when scalding had happened. The hot porridge should be removed as quickly as possible, i.e., within the first seconds, to limit the injury development.

The finding that the first few seconds are critical is also supported by research on beverage scalds, e.g., [17]. Following the porridge removal, even very fast tempered water application to the heated skin only resulted in minor injury reduction. Several studies do, however, conclude that prolonged tempered water cooling significantly reduces the burn severity, e.g., [10,15,30,31,32]. Though the long term moderate cooling process is not sufficiently understood by the international research community, scald burns should therefore be treated by prolonged, i.e., at least 20–30 min, of tempered water application [32]. It may simply be the case that tempered water application promotes the skin healing process. 

It could be hypothesized that some skin cells in a scalding or burn scenario become partly injured, i.e., to a point where continuous degrading versus gradual healing turns out to be temperature dependent. Or it could be hypothesized that live cells are attacked by chemical radicals from nearby damaged cells/cell tissue. If these damage reactions exhibit an Arrhenius type temperature dependency, could keeping the skin temperature at lower temperatures possibly halt further degradation processes? Could this favorable moderate temperature then rather stimulate the healing processes these first 20–30 minutes? Considerations like this are, however, outside the present models for skin injury modeling restricted to temperatures above 43 °C, and therefore, also outside the scope of the present work. Investigating such concepts could, however, represent an interesting topic for future research, as numerous studies conclude that 20–30 min tempered water application helps reduce the consequences of scalds and burns. 

There are several uncertainties regarding the modeling in the present work. The thermal properties of skin were assumed to be constant. They may, however, change with temperature. The temperature dependency of porridge thermal parameters, i.e. thermal conductivity, density, and specific heat, is taken from available literature data [20]. These values may, however, to some extent be dependent on the rice to milk ratio. Since the thermal conductivity of the porridge is higher than that of the skin, the contact temperature is more dependent on the lower thermal conductive solid in the contact zone, i.e., the skin. More water in the porridge would increase the thermal conductivity of the porridge some, but only slightly increase the porridge skin contact layer temperature. It could, however, result in lower viscosity, and thereby, less spill thicknesses. It should be noted that the properties modeled in the present work are realistic, but not necessarily worst-case scenarios.

The most important results in the present work are the benefit of serving porridge at lower temperatures and fast removal in scald scenarios. Though not thermally justified in the present work, prompt application of tempered water after porridge removal for skin cooling is documented by other researchers to be very beneficial [30]. Should a spill of hot porridge or similar food happen, it is demonstrated that it is of vital importance to get it promptly removed to mitigate skin injury development. Following the porridge removal, prolonged tempered water cooling is strongly advised to improve the post scald healing process.

## 5. Conclusions

The present work studied temperature development and thermal skin injury development for porridge spills to the skin. The porridge and skin temperatures were modeled numerically for selected initial porridge temperatures and spill layer thicknesses, as well as for spill removal and tempered water cooling. Pennes bioheat equation was used for calculating the corresponding skin injury (damage integral).

The porridge temperature and contact time turned out to be the most important injury parameters. For a 3 mm thick spill, an initial porridge temperature of about 70 °C seemed to be a limit for developing superficial partial-thickness burns. Increasing the initial temperature to 75 °C increased the skin injury significantly. At temperatures close to porridge processing temperature, severe skin injury developed nearly instantaneously.

It was demonstrated that prompt removal of the spilled porridge within the first seconds may significantly reduce skin injury. The general advice is, therefore, to avoid excessively hot porridge and, if spilled onto the body, to quickly remove the spill. Prompt cooling with tempered water at 15 °C was shown to have minor effect on the thermal skin damage. Prolonged tempered water cooling is, however, very much advised to improve post scald healing.

## Figures and Tables

**Table 1:** Properties of the involved skin layers (thermal conductivity, k, density, ρ and specific heat, C, are from [11]). Base case (Case A) epidermis thickness is marked in bold face.

Skin Layer	k (W/m·K)	ρ (kg/m^3^)	$C_{P}$ (J/kg·K)	a (m^2^/s)	Thickness (μm)
Epidermis	0.22	1200	3600	5.1 × 10^−8^	40, 50, **60**, 80
Dermis	0.40	1200	3600	9.3 × 10^−8^	0.002
Sub cutaneous	0.20	1000	2500	8.0 × 10^−8^	0.010
Muscle	0.45	1000	3800	1.2 × 10^−7^	0.030

**Table 2:** Damage integral (Ω) for selected cases.

Case	$L_{\mathrm{epi}}$ (μm)	$L_{\mathrm{porridge}}$ (mm)	$T_{\mathrm{porridge}}$ (°C)	$t_{\mathrm{off}}$ (s)	$t_{\mathrm{cool}}$ (s)	Ω	Comments
A	60	3.0	70	-	-	0.444	Base case, 60 μm epidermis, not removed
B	60	3.0	75	-	-	2.591	5 °C warmer than the base case (A)
C	60	3.0	65	-	-	0.075	5 °C colder than the base case (A)
D	50	3.0	70	-	-	0.491	50 μm epidermis
E	40	3.0	70	-	-	0.549	40 μm epidermis
F	60	2.0	70	-	-	0.206	2.0 mm porridge layer
G	60	4.0	70	-	-	0.642	4.0 mm porridge layer
H	60	3.0	70	10	-	0.103	Porridge removal at 10 s
I	60	3.0	70	10	20	0.103	Removal 10 s, water cooling t > 20 s
J	60	3.0	70	10	11	0.101	Removal 10 s, water cooling t > 11 s
K	60	3.0	70	20	-	0.239	Porridge removal at 20 s
L	60	3.0	70	30	-	0.338	Porridge removal at 30 s
M	60	3.0	75	10	-	0.599	75 °C, porridge removal 10 s
N	60	3.0	75	20	-	1.421	75 °C, porridge removal at 20 s
O	60	3.0	75	30	-	2.009	75 °C, porridge removal at 30 s
P	60	3.0	75	30	-	1.985	75 °C, removal at 30 s, water cooling t > 31 s
Q	60	3.0	75	10	11	0.588	75 °C, removal at 10 s, water cooling t > 11 s

## References

[1] He S., Alonge O., Agrawal P., Sharmin S., Islam I., Mashreky S.R., Arifeen S.E. (2017). Epidemiology of burns in rural Bangladesh: An update. Int. J. Environ. Res. Public Health.

[2] Burn Incidence and Treatment in the United States: 2016. http://www.ameriburn.org/resources_factsheet.php.

[3] Guillory A.N., Clayton R.P., Herndon D.N., Finnerty C.C. (2016). Cardiovascular dysfunction following burn injury: What we have learned from rat and mouse models. Int. J. Mol. Sci..

[4] Killat J., Reimers K., Choi C.Y., Jahn S., Vogt P.M., Radtke C. (2013). Cultivation of keratinocytes and fibroblasts in a three-dimensional bovine collagen-elastin matrix (Matriderm^®^) and application for full thickness wound coverage in vivo. Int. J. Mol. Sci..

[5] Rigo C., Ferroni L., Tocco I., Roman M., Munivrana I., Gardin C., Cairns W.R.L., Vindigni V., Azzena B., Barbante C. (2013). Active silver nanoparticles for wound healing. Int. J. Mol. Sci..

[6] Henriques F.C., Moritz A.R. (1947). Studies of thermal injury, I: The conduction of heat to and through skin and the temperatures attained therein: A theoretical and an experimental investigation. Am. J. Pathol..

[7] Moritz A., Henriques F.C. (1947). Studies of thermal injury, II: The relative importance of time and surface temperature in the causation of cutaneous burns. Am. J. Pathol..

[8] Moritz A.R. (1947). Studies of thermal injury, III: The pathology and pathogenesis of cutaneous burns: An experimental study. Am. J. Pathol..

[9] Fu M., Weng W.G., Yuan H.Y. (2014). Numerical simulation of the effects of blood perfusion, water diffusion, and vaporization on the skin temperature and burn injuries. Numer. Heat Transf. Part A Appl..

[10] Tobalem M., Harder Y., Tschanz E., Speidel V., Pittet-Cuénod B., Wettstein R. (2013). First-aid with warm water delays burn progression and increases skin survival. J. Plast. Reconstr. Aesthet. Surg..

[11] Johnson N.N., Abraham J.P., Helgeson Z.I., Minkowycz W.J., Sparrow E.M. (2011). An archive of skin-layer thicknesses and properties and calculations of scald burns with comparisons to experimental observations. J. Therm. Sci. Eng. Appl..

[12] Monds J.R., McDonald A.G. (2013). Determination of skin temperature distribution and heat flux during simulated fires using Green’s functions over finite-length scales. Appl. Therm. Eng..

[13] Buettner K. (1951). Effects of extreme heat and cold on human skin, II. Surface temperature, pain and heat conductivity in experiments with radiant heat. J. Appl. Physiol..

[14] Lawrence J.C., Bull J.P. (1976). Thermal conditions which cause skin burns. J. Eng. Med..

[15] Lau E.Y.K., Tam Y.-Y.M., Chiu T.W. (2016). Importance of clothing removal in scalds. Hong Kong Med. J..

[16] Log T. (2017). Skin temperatures of a pre-cooled wet person exposed to engulfing flames. Fire Saf. J..

[17] Abraham J.P., Plourde B., Vallez L., Stark J., Diller K.R. (2015). Estimating the time and temperature relationship for causation of deep-partial thickness skin burns. Burns.

[18] Abraham J.P., Nelson-Cheeseman B.B., Sparrow E., Wentz J.E., Gorman J.M., Wolf S.E. (2016). Comprehensive method to predict and quantify scald burns from beverage spills. Int. J. Hyperth..

[19] Abraham J.P., Hennessey M.P., Minkowycz W.J. (2011). A simple algebraic model to predict burn depth and injury. Int. Commun. Heat Mass Transf..

[20] Kadam S., Datta A.K. (2015). Estimation of Thermal Properties and Heat Transfer Study during Continuous Processing of Rice in Milk. Chem. Eng. Commun..

[21] Ng E.Y.K., Chua L.T. (2002). Prediction of skin burn injury, Part 2: Parametric and sensitivity analysis. Proc. Inst. Mech. Eng. H.

[22] Lipkin M., Hardy J.D. (1954). Measurement of some thermal properties of human tissues. J. Appl. Physiol..

[23] Rai K.N., Rai S.K. (1999). Heat transfer inside the tissues with a supplying vessel for the case when metabolic heat generation and blood perfusion are temperature dependent. Heat Mass Transf..

[24] Log T., Gustafsson S.E. (1995). Transient Plane Source (TPS) technique for measuring thermal transport properties of building materials. Fire Mater..

[25] Millington P.F., Wilkinson R. (1983). Skin.

[26] Viglianti B.L., Dewhirst M.W., Abraham J.P., Gorman J.-M., Sparrow E.M. (2014). Rationalization of thermal injury quantification methods: Application to skin burns. Burns.

[27] Henriques F.C., Moritz A. (1947). Studies of thermal injury, V: The predictability and the significance of thermal induced rate processes leading to irreversible epidermal injury. Arch. Pathol..

[28] Ye H., De S. (2017). Thermal injury of skin and subcutaneous tissues: A review of experimental approaches and numerical models. Burns.

[29] Log T. (2017). Modeling Skin Injury from Hot Spills on Clothing. Int. J. Environ. Res. Public Health..

[30] Vallez L.J., Plourde B.D., Wentz J.E., Nelson-Cheeseman B.B., Abraham J.P. (2017). A review of scald burn injuries. Intern. Med. Rev..

[31] Bourdon R.T., Nelson-Cheeseman B.B., Abraham J.P. (2016). Prediction, identification, and initial treatment guide for scald injuries. Aust. J. Emerg. Crit. Care Med..

[32] Bourdon R.T., Nelson-Cheeseman B.B., Abraham J.P. (2017). Review of the initial treatment and avoidance of scald injuries. World J. Dermatol..

